# Iatrogenic tibial arteriovenous fistula after Fogarty balloon catheter graft thrombectomy

**DOI:** 10.1002/ccr3.5050

**Published:** 2021-11-09

**Authors:** Spyros Papadoulas, Konstantinos Moulakakis, Natasa Kouri, Francesk Mulita, Andreas Tsimpoukis, Charalampos Seretis, Panagiotis Kitrou, Konstantinos Katsanos, Stavros K. Kakkos

**Affiliations:** ^1^ Department of Vascular Surgery University of Patras Medical School Patras Greece; ^2^ Department of Surgery University of Patras Medical School Patras Greece; ^3^ Department of Radiology University of Patras Medical School Patras Greece

**Keywords:** arteriovenous fistula, fogarty thrombectomy, graft thrombectomy, iatrogenic injury

## Abstract

A 75‐year‐old male presented with an immediately threatened grade IIb acute ischemia of the left leg due to thrombosis of a femoro‐infrapopliteal prosthetic bypass graft. After an urgent Computed Tomography Angiography, an urgent graft thrombectomy was performed using a 5 Fr Fogarty catheter, which had a troublesome distal passage, causing a tibial A‐V fistula.

## Graphical abstract

A hand‐held doppler device reaveals a continuous machinery bruit due to a tibial A‐V fistula

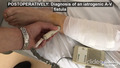

## 1 CASE DESCRIPTION

A 75‐year‐old man presented with an immediately threatened grade IIb acute ischemia of the left leg due to thrombosis of a femoro‐infrapopliteal prosthetic bypass graft. After an urgent computed tomography angiography, an urgent graft thrombectomy was performed using a 5 Fr Fogarty catheter, which had a troublesome distal passage. The leg recovered normal motor and sensory function. Surprisingly, a handheld Doppler device obtained a continuous machinery bruit in the anterior tibial artery/veins, suggesting an arteriovenous fistula, which was depicted in a subsequent digital subtraction angiography and a color duplex examination (Video S1). Most likely, the Fogarty catheter had unintentionally perforated the anterior tibial artery passing into one of the adjacent anterior tibial veins.^1^, ^2^ The patient was managed conservatively, and the arteriovenous fistula was found to be spontaneously thrombosed in a three‐month follow‐up (Video S1).

## CONFLICTS OF INTEREST

There are no conflicts of interest to declare.

## AUTHOR CONTRIBUTIONS

SP, KM, NK, FM, AT, and CS contributed to the clinical data collection and prepared the case report. SP, FM, PK, KK, and SK contributed to the design of the case report presentation and performed the final revision of the manuscript.

## ETHICAL APPROVAL

This report for a clinical image was in accordance with the Declaration of Helsinki.

## CONSENT

A formal informed consent was obtained from the patient prior to the publication of this article.

## Supporting information

Video S1

## Data Availability

Data are available on request from the authors.
